# Proteomics analysis of extracellular vesicles for biomarkers of autism spectrum disorder

**DOI:** 10.3389/fmolb.2024.1467398

**Published:** 2024-11-11

**Authors:** Houda Yasmine Ali Moussa, Kyung Chul Shin, Alberto de la Fuente, Ilham Bensmail, Houari B. Abdesselem, Janarthanan Ponraj, Said Mansour, Fouad A. Al-Shaban, Lawrence W. Stanton, Sara A. Abdulla, Yongsoo Park

**Affiliations:** ^1^ Neurological Disorders Research Center, Qatar Biomedical Research Institute (QBRI), Hamad Bin Khalifa University (HBKU), Qatar Foundation, Doha, Qatar; ^2^ Proteomics Core Facility, Hamad Bin Khalifa University (HBKU), Qatar Foundation, Doha, Qatar; ^3^ HBKU Core Labs, Hamad Bin Khalifa University (HBKU), Doha, Qatar; ^4^ College of Health and Life Sciences (CHLS), Hamad Bin Khalifa University (HBKU), Qatar Foundation, Doha, Qatar

**Keywords:** extracellular vesicle, biomarker, Olink, autism, ASD

## Abstract

**Background:**

Autism spectrum disorder (ASD) is a neurodevelopmental disorder characterized by symptoms that include social interaction deficits, language difficulties and restricted, repetitive behavior. Early intervention through medication and behavioral therapy can eliminate some ASD-related symptoms and significantly improve the life-quality of the affected individuals. Currently, the diagnosis of ASD is highly limited.

**Methods:**

To investigate the feasibility of early diagnosis of ASD, we tested extracellular vesicles (EVs) proteins obtained from ASD cases. First, plasma EVs were isolated from healthy controls (HCs) and ASD individuals and were analyzed using proximity extension assay (PEA) technology to quantify 1,196 protein expression level. Second, machine learning analysis and bioinformatic approaches were applied to explore how a combination of EV proteins could serve as biomarkers for ASD diagnosis.

**Results:**

No significant differences in the EV morphology and EV size distribution between HCs and ASD were observed, but the EV number was slightly lower in ASD plasma. We identified the top five downregulated proteins in plasma EVs isolated from ASD individuals: WW domain-containing protein 2 (WWP2), Heat shock protein 27 (HSP27), C-type lectin domain family 1 member B (CLEC1B), Cluster of differentiation 40 (CD40), and folate receptor alpha (FRalpha). Machine learning analysis and correlation analysis support the idea that these five EV proteins can be potential biomarkers for ASD.

**Conclusion:**

We identified the top five downregulated proteins in ASD EVs and examined that a combination of EV proteins could serve as biomarkers for ASD diagnosis.

## 1 Introduction

Autism spectrum disorder (ASD) is a complex neurodevelopmental condition, characterized by stereotyped repetitive behaviors and communication deficits (American Psychiatric Association, 2013). An increasing number of genetic variants implicated in ASD have been reported, suggesting a high degree of locus heterogeneity and contributions from rare and *de novo* variants (State and Levitt, 2011). Comorbidity is common in ASD, including attention-deficit hyperactivity disorder (ADHD) and epilepsy (Carter and Scherer, 2013). One of the major challenges in ASD research is to find reliable biomarkers that can help with early detection of ASD. Although some genetic factors have been linked to ASD risk, there is no definitive or consistent biomarker for ASD yet.

EVs are a group of vesicles surrounded by a lipid bilayer and are secreted by almost all cell types (Mulcahy et al., 2014). They mediate intercellular communication by transferring their contents horizontally (Veziroglu and Mias, 2020). EVs have critical functions in health and disease and offer potential clinical value as new biomarkers for early detection and therapeutic targets for treatment (Trino et al., 2021). EVs can cross the blood-brain barrier (BBB) (Alvarez-Erviti et al., 2011; Saeedi et al., 2019; Chen et al., 2016), there by circulating through the bloodstream. Since EVs mirror the cell and tissue of origin in terms of disease outcome and severity, their contents can serve as non-invasive biomarkers for various diseases (Trino et al., 2021; Huo et al., 2021) and plasma EVs can be used as biomarkers of neurological disorders (Ali Moussa et al., 2022).

EV proteins are promising liquid biopsy targets for early detection of Parkinson’s disease, as their profiles change in disease conditions (Kitamura et al., 2018). Three plasma EV proteins (clusterin, complement C1r subcomponent, and apolipoprotein A1) could serve as diagnostic biomarkers for Parkinson’s disease, and the expression of EV proteins is associated with disease progression (Kitamura et al., 2018). Plasma EV proteins could also help distinguish Alzheimer’s disease (AD) patients from healthy controls (Cai et al., 2022). However, no specific EV protein biomarkers have been yet identified for ASD.

In this study, we used size exclusion chromatography (SEC) to isolate EVs from the plasma of healthy controls (HCs) and ASD cases from Qatari and non-Qatari individuals living in Qatar. We then applied the proximity extension assay (PEA) Olink platform to analyze the EV proteome profiles. We identified the top five downregulated proteins in plasma EVs isolated from ASD cases and examined that a combination of EV proteins could serve as biomarkers for ASD diagnosis.

## 2 Materials and methods

### 2.1 Study cohort and blood collection

All procedures were performed under the approval of the Institutional Review Board (IRB# 2018-024) of Qatar Biomedical Research Institute (QBRI). The study cohort was obtained from QBRI’s Interdisciplinary Research Program (IDRP) depository and included plasma samples from 81 ASD and 26 healthy control (HCs) individuals, all residing in Qatar. The communication and social skills for the HCs were evaluated using the Social Communication Questionnaire (SCQ). ASD individuals were clinically diagnosed using the Diagnostic and Statistical Manual of Mental Disorders, Fifth Edition (DSM-5) criteria. Written informed consent and assent were obtained from all the HCs, ASD individuals and their surrogates. Demographical information of the participants is summarized in Table 1. Human peripheral blood samples were drawn from the HCs and ASD individuals into EDTA tubes. In the processing of blood samples, blood components were separated using density gradient centrifugation as previously described (Ali Moussa et al., 2022). The plasma supernatant was further centrifuged for 15 min to remove platelets and blood cells to obtain platelet-free plasma, which was aliquoted and stored at −80°C until further use for EV isolation.

**TABLE 1:** Participants’ demographical information.

	ASD cases	Healthy controls (HCs)
Number of participants	N = 81	N = 26
Age (Mean ± SD)	8.56 ± 2.17	11.08 ± 2.20
Gender (F/M)	17/64	13/13
ADOS-2 scores (Mean ± SD)	3.77 ± 1.7	—

### 2.2 Extracellular vesicles isolation

EVs were isolated from 250 µL of human plasma by size exclusion chromatography (SEC) using the qEVOriginal/35 nm columns (SP5, Izon Science, Christchurch, New Zealand). The plasma was thawed on ice and diluted with PBS for a final volume of 500 µL. It was then centrifuged at 3,000 g for 10 min and 10,000 g for 30 min to remove cell debris and large vesicles, followed by purification on the SEC columns. Fractions 1-25, including void volume were collected for some samples to verify the particle/protein profile. For the remaining samples, the void volume was discarded and only the high particle/low protein fractions were collected. EV-enriched fractions 2 and 3 with total volume of 1 mL were pooled and concentrated using pre-conditioned 100 kDa Amicon Ultra-15 centrifuge filters (UFC9100, Millipore) to a final volume of 170 µL. The amount of EV proteins was estimated by measuring the absorbance at 280 nm (A280). EV samples were aliquoted to minimize the freeze-thaw cycles and stored at −80°C until further analyzed.

### 2.3 Nanoparticle tracking analysis (NTA)

Particle size and particle number were determined using nanoparticle tracking analysis (NTA) (ZetaView, Particle Metrix, Germany). EV samples were diluted with filtered PBS to an average of 100 particles per frame and a final volume of 1 mL. Zetaview software (version 8.04.02 SP2) recorded particles at 11 camera positions and 30 frames per second.

### 2.4 Transmission electron microscopy (TEM)

Five microliter of EV suspension was deposited on carbon-coated 400-mesh copper grids (CF400-CU, Electron Microscopy Sciences) and incubated for 10 min. The EVs were then washed with ddH_2_O and excess fluid was absorbed with filter paper. Grids were negatively stained with uranyl acetate and embedded in methylcellulose-uranyl acetate. EVs were examined at 80 kV in Talos F200C Transmission Electron Microscope (Thermo Fisher Scientific). The images were acquired using bottom-mounted CETA camera.

### 2.5 Olink proximity extension assay

Protein profiling of EV samples was carried out using the proximity extension assay (PEA) from the Olink Target 96, testing a total of 1,196 proteins (13 panels including Neurology, Development, Neuro-exploratory, Inflammation, Immune Response, Cell Regulation, Organ Damage, Metabolism, Oncology II, Oncology III, Cardiometabolic, Cardiovascular II, and Cardiovascular III; Olink Bioscience, Uppsala, Sweden). A total of 1–10 × 10^7^ EV particles were subjected to each panel for PEA analysis. Following the standard protocol, the runs were performed by Olink-certified proteomics core facility at QBRI and were all validated by the Olink support team in Uppsala, Sweden. PEA is an ultrasensitive technology based on dual recognition of target proteins through matched pairs of antibodies labeled with DNA oligonucleotides (Assarsson et al., 2014). Quality control and data normalization were carried out using the Normalized Protein eXpression (NPX) software. Protein expression values were calculated as NPX; NPX is an arbitrary unit by Olink to quantify protein expression level on a log2 scale. Olink data that did not pass quality control were excluded from the analyses. NPX is a relative quantification unit that reflects the abundance of proteins in the sample and allows for comparison across different samples and conditions.

In the Olink assay, when two antibody pairs bind to their respective target protein, their attached oligonucleotides come into proximity, allowing a DNA polymerase to extend the oligonucleotides, forming a unique sequence. This sequence is then amplified and quantified using qPCR. The qPCR step produces a cycle threshold (Ct) value, which indicates the number of amplification cycles required to detect the target. A lower Ct value means higher protein concentration because fewer cycles are needed to reach the detection threshold. To ensure consistency across multiple plates, an internal control called the Interplate Control (IPC) is used. This helps to normalize Ct values across different plates, compensating for any technical variation between runs. The NPX value is calculated by taking the negative log2 transformation of the normalized Ct values. This transformation turns the relative quantification into a more interpretable scale where higher NPX values correspond to higher protein concentrations. NPX is expressed as follows.

$$\text{NPX}=-\log_{2}\;\left(\text{Normalized Ct}\right)$$

This conversion ensures that higher NPX values correspond to higher protein abundances, making it easier to compare protein levels across different samples.

### 2.6 Bioinformatics

The analysis for EV characterization experiments was conducted using GraphPad Prism software. Statistical analysis for proteomics data was performed using R software. Differentially expressed proteins were identified using the Limma (Linear Models for Microarray Data) package in R. Given the differences in the amount of EVs across samples, NPX of Olink data was normalized by incorporating EV particle numbers of each sample as a covariate in the linear model during statistical analysis to ensure accurate protein quantification. The model was designed to remove any bias introduced by different EV particle numbers.

The *p*-values of all the proteins were adjusted for multiple testing using the Benjamini–Hochberg (BH) method. The analytical model accounted for EV particle counts as influential covariates. Top differentially expressed proteins (TopDEPs) were selected based on a dual criterion: a fold change (FC ≥ 2) and a BH-adjusted *p*-value (adj *p*-value ≤ 0.05).

For Gene Ontology (GO) Enrichment Analysis, the R library clusterProfiler was used with focus on Cellular Components and Biological Process, and enriched GO terms passing adjusted *p*-values < 0.05 were identified. For Machine Learning, variable selection was performed using the MUVR, Boruta, and VSURF R packages, all set to their default parameters for optimal performance. The MLR3 library served as the foundation for training and evaluating an array of methods through repeated 4-fold cross validation. The MLR3 library also provides a function to create an ROC curve averaged over all validation folds and calculate the 95% Confidence Interval.

## 3 Results

### 3.1 Study cohort characteristics

We recruited 109 participants for our study, consisting of 81 ASD cases and 26 healthy controls (HCs) who were aged between 6 and 15 years. The mean ages for ASD cases and HCs were 8.56 ± 2.17 and 11.08 ± 2.20, respectively (Table 1). The HC group had an equal proportion of males and females (50%); however, the ASD group had a higher percentage of males (79%) due to the male predominance of ASD, which can be as high as 4:1 (Maenner et al., 2020). All ASD cases had a clinical diagnosis of ASD based on DSM-5 criteria and were evaluated using the ADOS-2 score. Table 1 shows the demographical information of ASD cases and HCs.

### 3.2 Characterization of EVs isolated from blood plasma of ASD and HCs

**FIGURE 1:**
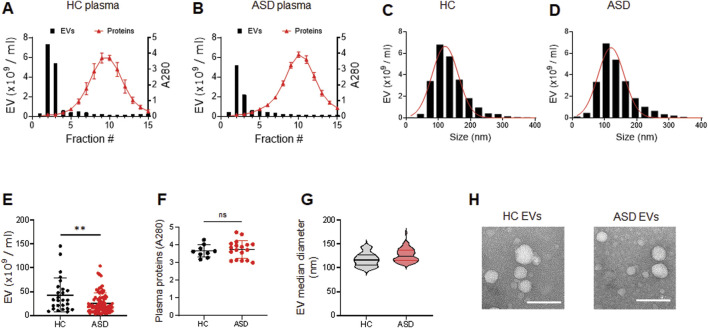
Characterization of plasma EVs isolated from healthy control (HC) and ASD individuals. **(A, B)** Representative elution profiles of plasma EVs and plasma proteins from HC **(A)** and ASD individuals **(B)**. EVs are the first to elute, followed by smaller protein complexes. Fractions 2 and 3 were pooled together as EV samples. EV particle numbers and protein concentration in each fraction were determined by NTA and the absorbance at a wavelength of 280 nm, respectively. **(C, D)** Representative size distribution of plasma EVs determined by NTA. Mean diameter (nm), 121.3 ± 40.32 SD for HC and 120.3 ± 40.53 SD for ASD. **(E)** EV particle numbers analyzed using NTA; HCs (n = 26) and ASD (n = 81). **(F)** Plasma protein concentration of HCs (n = 9) and ASD (n = 18). Total plasma proteins eluted in fractions 10 and 11 were measured based on absorbance at 280 nm. **(G)** Violin plots showing statistical median diameter (X50, nm) of EVs isolated from HC (n = 26) and ASD (n = 81) plasma; 117.6 nm ± 13.99 SD for HC and 126.1 nm ± 13.51 SD for ASD. **(H)** Morphological characterization of HC and ASD EVs using negative-stain transmission electron microscopy (TEM). Data in **(E, F)** are means ± SEM. Unpaired two-tailed t-test was used; **, *p* < 0.01.

We have optimized the protocol of plasma EV isolation using size exclusion chromatography (SEC), as previously described (Ali Moussa et al., 2022). The larger molecules elute first from the SEC column, followed by EVs, and plasma protein complexes are the last to elute (Figures 1A, B). We measured the particle number of EVs in each fraction by using nanoparticle tracking analysis (NTA). We combined fractions 2 and 3 as EV samples for higher purity, and also monitored the absorbance at 280 nm for protein elution profiles (Figures 1A, B); we used 0.25 mL plasma for this study. We confirmed that abundant plasma proteins are removed from EV samples to improve the purity (Ali Moussa et al., 2022) and soluble protein elution increases sharply from fraction 5; the elution profiles of EVs and plasma proteins obtained by SEC did not reveal any differences between HCs and ASD cases (Figures 1A, B). We then further analyzed the EV samples from fractions 2 and 3.

The plasma EVs were measured by NTA to determine their size distribution. The results showed that the plasma EVs from HCs and ASD had similar sizes, ranging from 50 to 200 nm; mean diameter (nm) of 121.3 ± 40.32 SD for HCs and 120.3 ± 40.53 SD for ASD (Figures 1C, D). Intriguingly, the number of EV particles was reduced in ASD plasma samples (Figure 1E), while the plasma protein concentration was similar between HCs and ASD samples (Figure 1F); total plasma proteins eluted in fractions 10 and 11 were measured based on absorbance at 280 nm. The median diameter of EVs did not differ between HCs and ASD (Figure 1G). The structure of HCs- and ASD-derived EVs characterized by atomic force microscopy (AFM) was comparable (Figure 1H). Overall, these data suggest that there were no significant differences in the morphology and size distribution, but the EV number was lower in ASD plasma.

### 3.3 EV protein profiling using the Olink platform

Olink analysis of 1,196 proteins demonstrated a distinct plasma EVs protein expression profile in individuals with ASD compared to HCs (see *Method* section for details). Differentially expressed proteins were identified using Limma package in R. Since the particle numbers of EVs in ASD plasma are slightly reduced (Figure 1E), a direct comparison of protein levels between HC and ASD EVs could be biased. Therefore, we normalized protein levels to account differences in the amount of EVs across samples to ensure accurate protein quantification. EV particle numbers for each sample were incorporated as a covariate in the linear model used for statistical analysis. This approach was implemented to eliminate potential bias arising from variations in EV particle counts across samples.

**FIGURE 2:**
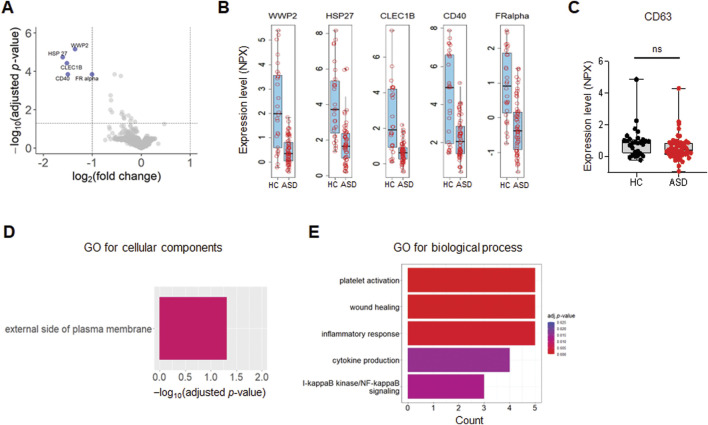
Differential expression of EV proteins. **(A)** The volcano plot of the differentially expressed (DE) proteins in plasma EVs isolated from HCs and ASD cases; log_2_ fold change (FC) against Limma −log_10_ BH-adjusted *p*-value. Color indicates significantly upregulated (red) and downregulated (blue) proteins with FC ≥ 2 and adjusted *p*-value < 0.05. **(B, C)** Box plots of the expression level presented as Olink’s normalized protein expression (NPX) for EV proteins **(B)** and CD63 **(C)** from control (n = 26) and ASD (n = 60). **(B)** Only five proteins are significantly downregulated with FC ≥ 2 and adjusted *p*-value < 0.05. **(D)** Gene Ontology (GO) enrichment analysis for cellular components of downregulated proteins in ASD EVs. The GO cut-off criteria included adjusted *p*-value < 0.05 and FC ≥ 1.5. **(E)** GO enrichment analysis for biological process of dysregulated proteins in ASD EVs. The GO cut-off criteria included *q* (adjusted *p* value) < 0.05 and 1.5 FC.

A list of the top differentially expressed proteins (TopDEPs) was summarized with a BH-adjusted *p*-value < 0.05 and fold change (FC) ≥ 2. A total of five downregulated proteins in ASD EVs were listed in the TopDEPs; no proteins were upregulated in ASD EVs (Figure 2A). Further details of all the significantly downregulated proteins are listed in Table 2. Top five downregulated proteins include WW domain-containing protein 2 (WWP2), Heat shock protein 27 (HSP27), C-type lectin domain family 1 member B (CLEC1B), Cluster of differentiation 40 (CD40), and folate receptor alpha (FRalpha) (Figure 2B).

**TABLE 2:** Predictive proteins using MUVR, Boruta, and VSURF.

Rank	Protein symbol	Protein full name	Gini impurity score	Fold change (FC)	Adjusted *p*-value
1	WWP2	WW domain containing E3 ubiquitin protein ligase 2	0.204	↓ 1.55	1.80 × 10^−7^
2	CD40	CD40 molecule	0.221	↓ 1.73	1.00 × 10^−5^
3	CLEC1B	C-type lectin domain family 1 member B	0.239	↓ 1.73	2.23 × 10^−6^
4	PAR1	Protease-activated receptor-1	0.257	↓ 0.66	1.51 × 10^−3^
5	HSP27	Heat shock protein 27	0.269	↓ 1.6	2.34 × 10^−5^
6	FRalpha	Folate receptor alpha	0.294	↓ 1.01	2.71 × 10^−4^

To further validate and characterize EVs, we analyzed CD63, a well-established marker protein for endosome-derived exosomes (Mathieu et al., 2021). While various tetraspanins, including CD9, CD63, and CD81, serve as general markers for EVs (Kowal et al., 2016), it is important to note that CD9 and CD81 are also present in ectosomes, which bud directly from the plasma membrane (Mathieu et al., 2021). Our previous findings indicated that >95% of plasma EV samples were positive for CD63, supporting that the majority of these EVs are indeed exosomes (Ali Moussa et al., 2022). In the current study, CD63 was included in the Olink proteomics panel, and expression level (NPX) of CD63 was high and comparable between HCs and ASD EV samples, with no significant differences observed (Figure 2C).

### 3.4 Gene ontology enrichment analysis of TopDEPs

We performed Gene Ontology (GO) enrichment analysis of TopDEPs to evaluate functional annotation of these downregulated proteins in ASD EVs. The cellular components in GO analysis only included external side of plasma membrane (Figure 2D), supporting that TopDEPs are derived from EVs. Top five significantly downregulated proteins are associated with EVs and can be potential biomarkers for ASD (see *Discussion*); WWP2, HSP27, CLEC1B, CD40, and FRalpha.

Next, we performed GO enrichment analysis of TopDEPs to identify the biological processes that were significantly dysregulated in ASD EVs (Figure 2E). We sorted the enriched biological terms by counts, which represent numbers of proteins associated with each term. The immune responses including inflammation and cytokine production were affected by downregulated proteins in ASD EVs (Figure 2E), implying that ASD EVs might be associated with immune dysregulation.

### 3.5 Machine learning to identify potential biomarkers

**FIGURE 3:**
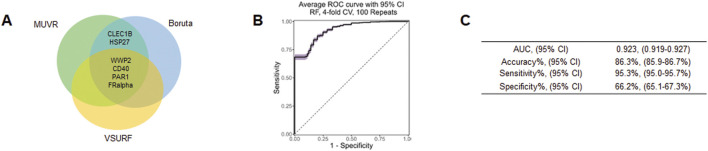
Machine learning outcome. **(A)** A Venn diagram of the overlapping proteins selected by MUVR, VSURF, and Boruta methods. **(B)** An ROC curve of the true positive rate versus the false positive rate for different threshold values of the classifier. **(C)** A table summarizing the performance metrics of the best classifier, which is the random forest using the intersect of the features selected by MUVR, Boruta and VSURF.

To further demonstrate potential biomarkers for ASD, we applied machine learning algorithms including minimally biased variable selection in R (MUVR), Boruta, and variable selection using random forests (VSURF) (Figure 3). We used three different feature selection methods to identify the most potential proteins for predicting the outcome, and then compared the performance of different classification algorithms. Six proteins overlapped between MUVR and Boruta, and four proteins were among MUVR, Boruta, and VSURF: WWP2, CD40, PAR1, FRalpha, CLEC1B, and HSP27 (Figure 3A). Details of six proteins are listed in Table 2; note that five out of these six proteins were also found to from the list of TopDEPs.

The diagnostic performance was tested using multiple multivariant supervised machine learning algorithms (random forest, generalized linear model, and support vector machines (SVM)). Six proteins were internally validated with four-fold cross-validations and 100 repeats (Figures 3B, C). The average ROC curve suggested that six proteins are strong candidates for diagnostic biomarkers for ASD with average AUC = 0.923, accuracy = 86.3%, sensitivity = 95.3%, specificity = 66.2% (Figure 3C).

## 4 Discussion

ASD affects approximately 1% of the global population, creating a significant public health burden in different communities including Qatar. According to our QBRI study on ASD (Alshaban et al., 2019), the prevalence of ASD in Qatar is 1.14% (one in every 87 children), leading to the financial burden and stress on parents and caregivers. Early intervention, whether through medication or behavioral therapy, can alleviate some ASD-related symptoms, significantly improving the life-quality of the affected individuals (Rogers et al., 2014; Dawson et al., 2012; Zwaigenbaum et al., 2009). Currently, early detection and intervention of ASD are highly limited and there are no medical kits or blood tests available for ASD diagnosis. Medical doctors can only check the child’s behavior and development to make a diagnosis of ASD, thereby limiting early intervention of ASD until kids become at least 4 or 5 years old. Early intervention and detection are critical to help ASD children effectively improve their language ability and social interaction.

In the literature, several genetic variants have been proposed as promising biomarkers for ASD (Nahas et al., 2024). Yet, because of the numerous gene mutations, ASD is extremely heterogenous and cannot be defined by unique polymorphisms. Other studies have identified differences in the microbiota and metabolic, immune, and nutritional markers, between control and ASD individuals (Lin et al., 2023; Chen et al., 2021; Edmiston et al., 2017). These potential biomarkers are all yet to be confirmed by large validation studies which can turn out to be extremely challenging. The various findings do, however, present valuable clues into the underlying molecular mechanisms and as to which biological processes are affected in ASD. In the present study, we have isolated and characterized plasma EVs in ASD and control individuals. We performed an extensive proteomics profiling, screening over 1,196 proteins, of which five are significantly downregulated in ASD EVs. To our knowledge, this study is the first and unique to investigate the EV protein cargo in ASD.

Due to the limited accessibility to the brain and cerebrospinal fluid (CSF) for biomarker discovery, blood is ideal for liquid biopsy, given its easier accessibility and non-invasive collection (Marrugo-Ramirez et al., 2018). EVs are very attractive diagnostic and therapeutic tools, particularly for brain disorders, because of their property to cross the BBB (Alvarez-Erviti et al., 2011; Saeedi et al., 2019; Chen et al., 2016). Thus, plasma EVs provide a potential therapeutic approach to neurological disorders. Brain-derived EVs might provide biomarkers for neuronal disorders, and EVs can be used in therapeutics as a drug delivery system to the brain (Yoo et al., 2018; Mustapic et al., 2017). EV proteins and RNA are considered promising biomarkers for neurodegenerative disease and neurodevelopmental disorders (Saeedi et al., 2019; Guix et al., 2018; Pulliam et al., 2019). Our data support that five EV proteins can pave the way for early diagnosis of ASD as novel biomarkers and have the potential to enhance diagnostic accuracy and facilitate earlier intervention strategies.

We applied stringent criteria to sensitively detect TopDEPs in ASD EVs, which could serve as potential biomarkers for ASD, using thresholds of FC ≥ 2 and an adjusted *p*-value ≤ 0.05. One of the major challenges in biomarker discovery is that many proteins lack specificity and selectivity, often resulting in large numbers of differentially expressed proteins, both upregulated and downregulated, in disease conditions. However, an abundance of candidate proteins does not necessarily facilitate biomarker identification. Notably, our study reveals that only five EV-associated proteins out of 1,196 proteins are selectively downregulated in ASD EVs, with no upregulated proteins detected. To ensure accurate protein quantification, we normalized NPX data to account for differences in EV particle numbers across samples by incorporating EV particle numbers as a covariate in the linear model for statistical analysis. Consequently, the observed differences in protein levels in our study reflect biological differences and variation in ASD rather than technical artifacts.

Top five significantly downregulated proteins are related to EV biogenesis, function and signaling: 1) WWP2, an E3 ubiquitin ligase, regulates EV release by ubiquitination of EV proteins (Nabhan et al., 2012); 2) HSP27 is a heat shock protein, which is elevated in the blood in various diseases (Reddy et al., 2018) and extracellular HSP27 may have functions in pathological conditions (De Maio and Vazquez, 2013). HSP27 is present in EVs released from THP-1 cells (Shi et al., 2019) and can be transferred to recipient cells via EVs (Reddy et al., 2018); 3) CLEC1B is a receptor involved in transmembrane signaling (Huysamen and Brown, 2009) and is highly expressed in neuron-derived exosomes (Pulliam et al., 2019); 4) CD40 is a protein present in plasma EVs from non-Hodgkin lymphoma patients (Martinez et al., 2022) and tumor-derived EVs (Hagerbrand et al., 2022), suggesting its potential as a cancer biomarker; 5) FRalpha is present in EVs and involved in folate transport into the brain through EVs (Grapp et al., 2013). Altogether, our data support that these five proteins may serve as useful EV biomarkers for ASD diagnosis.

Among the proteins we show to be downregulated in ASD individuals is HSP27 which is thought to have major protective effects against many cellular stresses (Latchman, 2005). This was in accordance with a previously published study evaluating protein levels in the blood of ASD children and found HSP27 to be decreased (Tsukurova, 2018). Over-expression of HSP27 has been shown to protect and rescue neuronal and non-neuronal cells from cell damage and death (Latchman, 2005; Dave et al., 2023). HSP27 is a biomarker candidate for neurodegenerative diseases like Alzheimer’s disease, because HSP27 is neuroprotective by protecting neurons from protein misfolding and oxidative stress (Wilhelmus et al., 2006). CD40 is important in neuroinflammation and is a potential biomarker for Alzheimer’s disease (Kim et al., 2023). FRalpha is involved in folate transport, and disruptions in folate metabolism have been linked to autism (Ramaekers et al., 2007).

While we have included key covariates that directly impact EV quantification, such as EV particle numbers, additional factors that might act as confounders—such as age, gender, or disease severity—were not explicitly considered in the current analysis. These confounding factors could potentially affect the relationship between protein levels and disease condition. To further refine our analysis and minimize the influence of these confounders, future studies could incorporate additional covariates based on clinical and demographic data.

The percentage of brain-derived EVs in plasma is typically low, given that most EVs circulating in the bloodstream originate from immune cells (Yanez-Mo et al., 2015; Thery et al., 2018). Noteworthy is the connection of the five TopDEPs identified in ASD EVs with immune responses and cytokine production (Figure 2E). This suggests that ASD EVs may play a role in modulating chronic inflammation. While chronic inflammation and immune dysregulation have been proposed as potential contributors to the characteristic features of autism (Arteaga-Henriquez et al., 2023), the mechanisms by which ASD EVs regulate chronic inflammation remain to be elucidated in further studies.

## Data Availability

The raw data supporting the conclusions of this article will be made available by the authors, without undue reservation.

## Abbreviations

ASD, Autism spectrum disorder; AFM, Atomic force microscopy; AD, Alzheimer’s disease; ADHD, Attention-deficit hyperactivity disorder; BBB, Blood-brain barrier; CLEC1B, C-type lectin domain family 1 member B; CD40, Cluster of differentiation 40; DSM-5, Diagnostic and Statistical Manual of Mental Disorders, Fifth Edition; EVs, Extracellular vesicles; Fralpha, Folate receptor alpha; FC, Fold change; GO, Gene Ontology; HCs, Healthy controls; HSP27, Heat shock protein 27; MUVR, Minimally biased variable selection in R; NTA, Nanoparticle tracking analysis; NPX, Normalized Protein eXpression; PAR1, Protease-activated receptor-1; PEA, Proximity extension assay; SEC, Size exclusion chromatography; SVM, Support vector machines; TopDEPs, Top differentially expressed proteins; VSURF, Variable selection using random forests; WWP2, WW domain-containing protein 2.
